# The Training and Evaluation of the “Dual-Index” Screening Method for Neonatal Congenital Heart Disease: A Multi-Center Study in China

**DOI:** 10.3390/ijns11010008

**Published:** 2025-01-14

**Authors:** Panpan Huang, Qing Gu, Xiaoting Zhu, Ijaz ul Haq, Liling Li, Xiaojing Hu, Guoying Huang

**Affiliations:** 1 Fujian Key Laboratory of Neonatal Diseases, Xiamen Children’s Hospital (Children’s Hospital of Fudan University at Xiamen), Xiamen 361006, China; 2 Xiamen Key Laboratory of Neonatal Diseases, Xiamen Children’s Hospital (Children’s Hospital of Fudan University at Xiamen), Xiamen 361006, China; 3 Children’s Hospital of Fudan University, Shanghai 201102, China; qinggu7633@163.com (Q.G.);; 4 National Management Office of Neonatal Screening Project for CHD, Shanghai 201102, China; 5 Shanghai Key Laboratory of Birth Defects, Shanghai 201102, China; 6 Innovation Unit of Early Prevention and Control of Genetically Related Cardiovascular Diseases in Children, Chinese Academy of Medical Science (2018RU002), Beijing 100700, China

**Keywords:** congenital heart disease, “dual-index” method, newborn, screening, training

## Abstract

Background: This study aimed to enhance the scope of neonatal congenital heart disease (CHD) screening by evaluating the effectiveness of training personnel in CHD screening using the “dual-index” method, combining pulse oximetry with cardiac murmur auscultation. Methods: From 2019 to 2022, a total of 2374 screening personnel from the Xinjiang, Yunnan, Hainan, Fujian, and Anhui provinces underwent training in neonatal CHD screening using the “dual-index” method, which involves pulse oximetry and cardiac murmur auscultation. Pre- and post-training assessments were conducted using a neonatal CHD screening knowledge questionnaire, distributed through the Questionnaire Star platform, to evaluate the impact of the training. The annual neonatal CHD screening rates were consistently recorded in these five provinces during the same period to assess the increase in screening coverage. Results: After the training, the screening personnel exhibited a significantly improved understanding of the neonatal CHD screening method (*p* < 0.001). Additionally, the professional background (*t* = −8.007, *p* < 0.001) and years of experience (*t* = 2.839, *p* = 0.005) of the screening personnel were identified as independent factors influencing their screening knowledge. During the same period, there was consistent linear growth in the screening coverage rate for neonatal CHD across the five provinces (*χ*^2^ = 121065.416, *p* < 0.001). Conclusion: Standardized training in the “dual-index” method, incorporating pulse oximetry and cardiac murmur auscultation, for screening personnel significantly enhances their screening knowledge, thereby playing a critical role in expanding the coverage of neonatal CHD screening.

## 1. Background

Congenital heart disease (CHD) comprises a group of structural or hemodynamic heart abnormalities and ranks among the most prevalent birth defects [1,2,3,4]. It affects approximately 6 to 11 out of every 1000 live births [5,6,7,8]. These conditions contribute to 10% of infant mortality and 46% of deaths associated with congenital malformations [9]. Critical congenital heart disease (CCHD) represents around 25% of all CHD cases [10]. Studies [11] in Western countries have shown that the overall misdiagnosis rate of asymptomatic CCHD without a prenatal diagnosis ranges from 12% to 50%. In China, when the same evaluation method is employed, the misdiagnosis rate is as high as 71.02% [12]. The rate of disability and mortality is significantly linked to missed diagnoses or delayed treatment [13]. Pulse oximetry (POX) stands out as a reliable indicator for early CHD screening [14,15]. Professor Guoying Huang’s research group has developed a novel “dual-index” screening method that integrates pulse oximetry with cardiac murmur auscultation for the early detection of congenital heart disease (CHD) in neonates within 6 to 72 h after birth. This innovative approach has significantly enhanced the sensitivity and specificity of screening for CCHD, achieving a remarkable level of 91.22% sensitivity and 98.89% specificity [13]. China also adopted the “dual-index” screening method for newborn CHD screening in July 2018 [16]. However, in the “dual-index” screening program, whether it involves POX measurement technology or heart murmur auscultation technology, screening personnel need to undergo training and continuous learning to ensure standardization and accuracy. The American Academy of Pediatrics recommends that screening for critical congenital heart disease (CCHD) should be performed by individuals who possess pulse oximetry testing within their professional scope, are trained in the application of pulse oximetry and the CCHD algorithm, and routinely utilize pulse oximetry for various clinical purposes [10]. The prevalence of CHD varies greatly among different regions in China. In addition to the diversity of newborn living environments and genetic factors, differences in the skills of CHD screeners also affect the detection rate of CHD. Similarly, the low rate of CHD diagnoses among screening-positive newborns in CHD diagnostic centers also suggests the insufficient capacity of screening personnel [17]. Therefore, in order to ensure the homogeneity of neonatal CHD screening, we carried out multi-center training and summarized the training’s effectiveness and planned to find a suitable training program for CHD screening in developing countries.

## 2. Methods

### 2.1. Study Design and Participants

Between 2019 and 2022, a total of 2374 screening personnel, selected from the Xinjiang, Yunnan, Hainan, Fujian, and Anhui provinces, underwent comprehensive training in neonatal CHD screening. This training incorporated the “dual-index” methodology, combining pulse oximetry and cardiac murmur auscultation. The subsequent analysis focused on the impact of this training, while the annual coverage rate of the neonatal CHD screening in these five provinces was consistently monitored, with prenatally diagnosed cases of CHD being excluded.

### 2.2. Procedures

Personnel from the National Management Office of the Neonatal Screening Project for CHD at the Children’s Hospital of Fudan University conducted training in the aforementioned five provinces: Xinjiang, Yunnan, Hainan, Fujian, and Anhui, which have a medical cooperation relationship. The training was conducted through both centralized online and offline teaching methods, ensuring that each province received at least one session of either online or offline training. Multimedia techniques, such as case studies, visual presentations, video demonstrations and interactive teaching, were used for effective knowledge dissemination. The specific training content is detailed in Table 1, outlining the “dual-index” screening method for neonatal CHD.

The training questionnaire was designed by the personnel of the National Management Office of the Neonatal Screening Project for CHD, drawing on research conducted by Donna [18]. The questionnaire comprised two sections: a general information survey and ten multiple-choice questions. The general information survey collected details about the screening personnel’s occupation, department, professional title, years of experience, and city. The ten multiple-choice questions covered key knowledge points, including factors influencing pulse oximetry, the appropriate timing of screening, and criteria for identifying positive screening results. All of these elements were included in the training program. The questionnaire had a total score of 50 points, with each multiple-choice question accounting for 5 points. The higher the score, the better the screening knowledge of the screening personnel. The questionnaire was administered both before and after the training, distributed using the Questionnaire Star platform. The pre-test allowed open questions to be answered before training and was closed after 5 min. The post-test was accessible after the training and was closed 5 min later. Before answering, the training participants received an explanation of the questionnaire and provided informed consent. After each response, the personnel reviewed the authenticity and the completeness of each questionnaire, eliminating invalid submissions. A total of 2374 effective pre-test questionnaires and 2101 effective post-test questionnaires were collected, resulting in an effective recovery rate of 88.50%.

In July 2018, the National Health Commission introduced the Work Plan for the Newborn Congenital Heart Disease Screening Program as a public health initiative. This program was implemented in 24 provinces, including municipalities and districts such as Shanghai, Hainan, and Yunnan. The relevant personnel gathered information about newborns and recorded it in the information management system to establish the national CHD screening program database. The screening data for newborns born between 2019 and 2022 in Xinjiang, Yunnan, Hainan, Fujian, and Anhui provinces were extracted from this database. However, there is a scarcity of data from 2018, as this program was initiated in that year.

The assessment of the training’s effectiveness involved tallying the scores from the pre-training and post-training questionnaires completed by the screening personnel and calculating the correct answer rates for each item in the questionnaire. Additionally, factors influencing the knowledge of the screening methods for neonatal CHD among the screeners were analyzed. To gauge the coverage rate of the neonatal CHD screening in these five provinces during the same period, the screening rate was determined using the following formula: Screening coverage rate (%) = (Number of infants screened/Number of live births) × 100%.

### 2.3. Statistical Analysis

The general information about the screening personnel was presented in terms of the number and composition ratio. The correct answer rate for each item in the training questionnaire and the screening coverage rate for neonatal CHD were compared using the Chi-square test. To determine if there was a year-by-year linear trend in the screening rate for CHD among newborns from 2019 to 2022, we employed the trend Chi-square test. For measurement data, such as training questionnaire scores, descriptive statistics were used, presenting the data as mean ± standard deviation. The comparison of scores before and after training was conducted using the *t*-test. Furthermore, we employed a multiple linear regression analysis to explore the factors influencing the screeners’ mastery of neonatal CHD screening knowledge. All the statistical tests were two-tailed, and the significance was set at *p* < 0.05.

## 3. Results

### 3.1. Training Effect

A total of 2374 questionnaires were gathered during the pre-test and 2101 questionnaires were gathered during the post-test. The comprehensive information pertaining to the screening personnel who participated in the training is presented in Table 2.

The questionnaire score of the screening personnel prior to training was (36.04 ± 9.67), and following the training, it was (44.09 ± 8.78). A statistically significant difference was observed in the questionnaire scores of the screening personnel before and after the training (*t* = −29.163, *p* < 0.001).

The screening personnel’s knowledge of neonatal CHD screening before and after the training is shown in Table 3. Before the training, it was evident that there were notable deficiencies in the screening personnel’s understanding of neonatal CHD screening methods, as reflected by their questionnaire responses. Following the training, there was a general improvement in the screening personnel’s correct rates for all the questionnaire items. A significant difference in the neonatal CHD screening methods before and after training was observed (*p* < 0.001). However, the screening personnel’s assessment of positive screening results were not accurate enough.

### 3.2. Analysis of Factors Affecting Screeners’ Mastery of Neonatal CHD Screening Knowledge

Statistically significant differences were observed in the questionnaire scores for newborn CHD screening among the screening personnel based on their occupations, departments, years of experience, and professional titles, as indicated in Table 4.

The scores obtained by the screening personnel on the CHD screening questionnaire were utilized as the dependent variable. Items that exhibited statistically significant differences in the univariate analysis were designated as independent variables for conducting a multiple linear regression analysis. The mode of assignment for independent variables was as follows: occupation (physician = 1, nurse = 2), department (neonatology = 1, pediatric internal medicine = 2, administrative departments = 3, obstetrics = 4, pediatric surgery = 5), seniority (≤3 years = 1, 3–5 years = 2, 5–10 years = 3, 10–20 years = 4, ≥20 years = 5), and professional title (primary = 1, intermediate = 2, deputy senior = 3, senior = 4). The department and seniority of the screeners emerged as independent factors that significantly influenced the screeners’ mastery of neonatal CHD screening knowledge, as outlined in Table 5.

### 3.3. The Evaluation of the Effect of Training on the Coverage Rate of Neonatal CHD Screening

**Figure 1:**
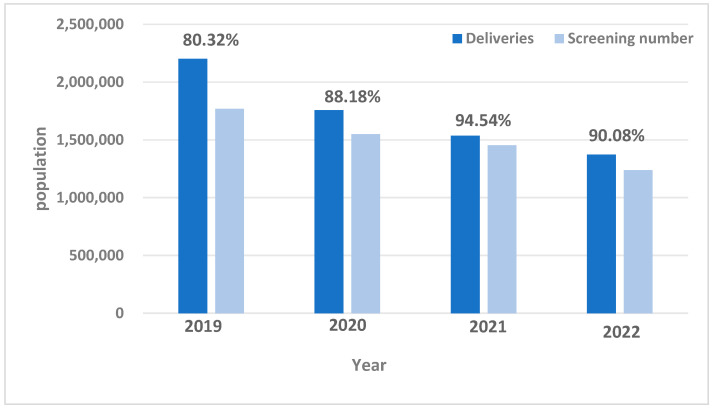
Screening rate of neonatal CHD in 5 provinces during 2019–2022. Note: Screening rate (%) = (Number of infants screened/number of live births) × 100%.

The trend Chi-square test was applied to scrutinize the CHD screening rate among newborns across five provinces. The analysis revealed a consistent year-by-year linear increase in the screening rate of neonatal CHD in the provinces of Xinjiang, Yunnan, Hainan, Fujian, and Anhui from 2019 to 2022, with a substantial linear upward trend (*χ*^2^ = 121,065.416, *p* < 0.001). The screening rate of neonatal CHD in the five provinces during 2019–2022 are shown in Figure 1. Although the screening rate of the five provinces showed an overall upward trend, it decreased slightly in 2022 compared with 2021, which was caused by the closure of some cities during the COVID-19 epidemic.

## 4. Discussion

### 4.1. On-the-Job Training Can Improve the Screening Personnel’s Knowledge of Neonatal CHD Screening

Following the training, the neonatal CHD questionnaire scores of the screening personnel increased from (36.04 ± 9.670) to (44.09 ± 8.784), with a statistically significant difference observed before and after the training (*t* = −29.163, *p* < 0.001). Simultaneously, the accuracy of the screeners’ responses to the questionnaire also exhibited a general improvement, signifying enhanced mastery of the “dual-index” neonatal CHD screening content in comparison to the pre-training levels (*p* < 0.001). Donna [18] conducted a continuous quality improvement program in nursing, offering online nursing education training to maternity hospital nurses. The results revealed a significant increase in the number of correct responses immediately after training (9.1 ± 1.0) compared to the baseline (8.4 ± 1.2) (*t* = 3.02, *p* = 0.004). The Texas Pulse Oximetry Project nurse educators and hospital nurse champions conducted an accredited, 1 h training session for 215 nurses at fifteen hospitals in South Texas. The nurses’ knowledge assessment improved from 71 to 92.5% (*p*< 0.001) [19]. The findings of this study indicate that on-the-job training can enhance the knowledge of screening personnel regarding neonatal congenital heart disease (CHD) screening. The screening personnel do not have a thorough understanding of the positive judgment of newborn screening results, suggesting that future training should focus on helping screeners understand the positive judgment of screening results.

### 4.2. The Professional Background and Experience of Screening Personnel Are Independent Factors That Significantly Influence the Screeners’ Mastery of Neonatal CHD Screening Knowledge

The findings of this study underscore the significance of the professional background and seniority of screeners as independent factors influencing the screeners’ mastery of neonatal CHD screening knowledge. Notably, personnel in the neonatology department achieved significantly higher questionnaire scores than their counterparts in other departments. (*t* = 13.046, *p* < 0.001). Neonatal oxygen saturation is the percentage of oxygenated hemoglobin bound by oxygen in the blood to the total amount of hemoglobin bound. This disparity can be attributed to the critical role of oxygen saturation (SpO_2_) as a direct indicator of neonatal oxygenation, enabling the timely detection of hypoxemia among newborns. It is of great significance to guide neonatal cardiopulmonary resuscitation [20]. A pulse oximeter is often used in clinic to monitor the pulse oximetry of newborns noninvasively and continuously to observe the condition of newborns. Given that the vast majority of infants admitted to the neonatal unit require SpO_2_ monitoring during hospitalization, it follows that screening personnel in the neonatology department possess greater expertise in neonatal care, resulting in higher questionnaire scores. Within the four age groups (≤3 years, 3–5 years, 5–10 years, and 10–20 years), the awareness of newborn CHD screening among the screening personnel increased with their years of service. These increased years correspond to more learning and training opportunities, the accumulation of professional knowledge, and the development of a broader skill set, ultimately contributing to enhanced work proficiency and more comprehensive decision making.

### 4.3. Effective Training Can Improve the Screening Coverage Rate of Neonatal CHD

The Healthy Children Action Plan (2021–2025) issued by the National Health Commission in 2021 mandates that newborn CHD screening should cover all districts and counties, with a screening rate exceeding 60% [21]. Shanghai, as a leading Chinese city, achieved a remarkable 96.9% screening coverage rate for newborns with CHD in 2017 [22]. In this study, post-training, the screening rate for neonatal CHD across the five provinces during 2019–2022 consistently exceeded 60%, demonstrating a promising upward trend. This highlights the importance of scaling up training initiatives to improve the national coverage of neonatal CHD screening. Effective training can also contribute to a reduction in the false positive rate. After conducting two extensive multi-center studies, scholar Xiaojing Hu [23,24] found that training substantially reduced the false positive rate of CCHD screening, decreasing it from 2.50% to 1.20%. The improvement of screening coverage means that more children with CHD can be detected and treated early. As a result, mortality due to congenital heart disease decreased. Since June 2016, the Shanghai Municipal Health and Family Planning Commission has included neonatal CHD screening into the routine neonatal screening program, and standardized screening training has been conducted for medical staff in all 86 midwifery institutions in Shanghai. In July 2018, the “dual-index” screening method was promoted nationwide. By the end of 2019, 28 provinces in China had launched screening programs for CHD in newborns. After the introduction of routine CHD screening in newborns in Shanghai, the infant mortality rate dropped from 4.58‰ to 2.30‰ from 2015 to 2021, and the mortality rate of children under 5 years old with CHD dropped from 25.93 percent to 16.61 percent [25]. From 2004 to 2018, 15 969 children aged 0 to 1 years died of CHD in China, and the standardized mortality rate of CHD in children aged 0 to 1 years decreased from 106.81/100,000 to 38.70/100,000 [26]. As a public health intervention measure, CHD screening really embodies the significance of tertiary prevention.

### 4.4. Initial Training Should Be Carried out and Regularly Updated for Screening Personnel Before the Promotion of Neonatal CHD Screening

The “Dual-index” screening method includes cardiac murmur auscultation and POX determination. As a painless and noninvasive test, POX screening offers a cost-effective and convenient alternative to other clinical screening methods for neonatal diseases, such as hypothyroidism. However, it is important to note that while POX screening is relatively simple to conduct, the correct screening technique is crucial to ensure accurate results. The accuracy of pulse oximeters can be influenced by motion artifacts, poor perfusion, and electromagnetic interference [27,28,29]. The auscultation of heart sounds in neonates with CHD has the characteristics of misdiagnosis, missed diagnosis and poor homogeneity. The cardiac murmur of newborns is different from that of children and adults, the medical staff require additional training to master the necessary techniques [13]. Therefore, initial training should be carried out before the promotion of neonatal CHD screening. Neonatal CHD screening has been included in the routine neonatal screening program in Shanghai since 2016. By December 2021, a total of 2294 medical staff had been trained and obtained neonatal CHD screening certificates [25]. The training and on-site supervision of medical staff and delivery facilities are conducted at least twice a year. During the training, the quality control center introduced the screening strategy and referral system for neonatal CHD in detail to ensure the accuracy and reliability of screening results and data registration. In the TEXAS educational and quality improvement project [19], when screening results indicate missed or inappropriate screening, hospital nurse champions provide additional education as a measure for quality improvement. They also offer one-on-one training to nurses in the ward as needed. The screening data of Hainan Province in China from 2019 to 2021 showed that the CHD diagnosis rate of screening-positive people was 12.57%, while in China’s first-tier cities, such as Shanghai (2017 to 2021), it was 21.47%. The low screening-positive diagnosis rate means that screening skills are insufficient, and screening personnel need more training. Therefore, initial training should be carried out and regularly updated for screening personnel before the promotion of neonatal CHD screening.

## 5. Limitations

The assessment of heart murmurs through auscultation is susceptible to the experience of physicians. However, the ten multiple-choice questions in this questionnaire are exclusively focused on topics related to pulse oximetry (POX). They do not encompass an evaluation of heart murmur auscultation. Furthermore, comprehensive data regarding the screening personnel were not collected. Consequently, the assessment of factors influencing the knowledge of neonatal CHD screening among screening personnel lacks the desired accuracy.

## 6. Conclusions

The prompt identification of congenital heart disease plays a pivotal role in improving the neonatal prognosis and mitigating the adverse consequences associated with delayed treatment. Effective training has been shown to significantly enhance the knowledge of screening personnel in neonatal CHD screening methods, facilitating the seamless implementation of screening.

## Figures and Tables

**Table 1:** “Dual-index” screening method for neonatal CHD.

Theme	Content
What is the “dual-index” screening method	① Which two indicators are specifically referred to in the “dual- index” screening method? ② Why choose these two indicators? ③ Operation video of “dual- index” screening. ④ Screening tools for “dual- index” screening. ⑤ Classification and epidemiology of congenital heart disease
Auscultation of heart murmur	① Cardiac valve auscultation area. ② Normal heart sound. ③ The mechanism of a cardiac murmur. ④ The location of the heart murmur. ⑤ Duration of heart murmur. ⑥ Loudness of heart murmur. ⑦ Differential diagnosis of heart murmur. ⑧ Murmurs alone do not have a high positive predictive value in detecting CHD.
Measurement of pulse oximetry	① Measurement principle of pulse oximetry. ② Influencing factors of pulse oximetry. ③ Clinical pulse oximetry measurement tools. ④ Methods and precautions for determination of pulse oximetry in CHD screening
Judgment of screening results	When screening for congenital heart disease in newborns 6–72 h after birth, the judging criteria are (1) Negative: the heart sound was below grade 2/6, POX of either limb ≥ 95%, POX of difference of upper and lower limb < 3%. (2) Positive: the heart sound is level 2/6 and above and meets any of the following 3: ① POX of right hand or either foot < 90%. ② POX is 90–94% in both right hand and either foot for 2 consecutive times (interval 2–4 h). ③ The difference values of POX in both right hand and either foot for 2 consecutive measurements (interval 2–4 h) were >3%.

**Table 2:** General information of the screening personnel who participated in the training.

		Pre-Test, *n* (%) (*n* = 2374)	Post-Test, *n* (%) (*n* = 2101)
occupation	Physician;	1415 (59.60)	1231 (58.60)
	Nurse.	959 (40.40)	870 (41.40)
department	Obstetrics;	1398 (58.89)	1274 (60.64)
	Pediatric internal medicine;	358 (15.08)	334 (15.90)
	Administrative departments such as the Medical Affairs Department;	335 (14.11)	247 (11.76)
	Neonatology;	225 (9.48)	208 (9.90)
	Pediatric surgery.	58 (2.44)	38 (1.80)
seniority	≤3 years;	506 (21.31)	437 (20.80)
	3~5 years;	384 (16.18)	324 (15.42)
	5~10 years;	518 (21.82)	477 (22.70)
	10~20 years;	512 (21.57)	453 (21.56)
	≥20 years.	454 (19.12)	410 (19.52)
professional title	Primary;	1331 (56.07)	1214 (57.78)
	Intermediate;	649 (27.34)	557 (26.51)
	Deputy senior;	286 (12.05)	258 (12.28)
	Senior.	108 (4.54)	72 (3.43)

**Table 3:** Screening personnel’s knowledge of neonatal CHD screening before and after training.

Item	Pre-Training (*n* = 2374)	Post-Training (*n* = 2101)	Statistics	*p*
	Correct Number (%)	Correct Number (%)		
Do movement, crying, cold extremities or shivering, bilirubin lamps and surgical lights affect the accuracy of the pulse oximetry (pulse ox) reading?	2107 (88.75)	2013 (95.81)	76.029	<0.001
One clean, disposable pulse ox probe can be used on up to 5 patients.	2178 (91.74)	1992 (94.81)	16.520	<0.001
Placing the pulse ox probe on the same extremity that you are taking the blood pressure, performing the pulse ox test while the infant is crying, using a clip on the finger of an infant, infant skin color or jaundice, all of the following can affect the accuracy of the pulse ox reading except.	1271 (53.54)	1762 (83.86)	469.361	<0.001
Pulse ox screening will detect all forms of CHD.	2048 (86.27)	1948 (92.71)	48.511	<0.001
The screening guidelines state that pulse ox should be performed on.	1540 (64.87)	1857 (88.39)	337.095	<0.001
Pulse ox screening should be performed when the infant is of what age?	1517 (63.90)	1810 (86.15)	289.282	<0.001
Pulse ox readings are greater than 94% for both right hand and right foot and there is greater than a 3% difference between the 2nd and 3rd measures each separated by 1 h, should be reported ?	1483 (62.47)	1891 (90.00)	455.596	<0.001
The first screen the upper extremity (UE) sat is 100% and lower extremity (LE) saturation is 96%, please determine whether the answer is pass or fail.	1587 (66.84)	1654 (78.72)	78.703	<0.001
Upper extremity saturation is 99% and lower extremity sat is 98%, please determine whether the answer is pass or fail.	1812 (76.33)	1836 (87.39)	90.502	<0.001
Upper extremity sat is 89% and lower extremity sat is 87%, please determine whether the answer is pass or fail.	1569 (66.09)	1762 (83.86)	185.038	<0.001

**Table 4:** Comparison of the scores of neonatal CHD screening questionnaire among screening personnel with different characteristics (*n* = 2374).

Item	Score (x ± s)	*t*/*F*	*p*
Occupation		2.936	0.003
physician	36.52 ± 9.38		
nurse	35.33 ± 10.05		
Department		13.046	<0.001
neonatology	39.74 ± 8.70		
pediatric internal medicine	38.24 ± 8.80		
administrative departments	36.36 ± 9.75		
obstetrics	35.49 ± 9.96		
pediatric surgery	34.29 ± 9.76		
Seniority		13.468	<0.001
≤3 years	34.01 ± 10.77		
3~5 years	35.59 ± 9.79		
5~10 years	36.91 ± 9.22		
10~20 years	37.97 ± 9.05		
≥20 years	37.13 ± 9.42		
Professional title		24.189	<0.001
primary	34.93 ± 10.25		
intermediate	38.47 ± 8.60		
deputy senior	37.84 ± 8.39		
senior	41.63 ± 8.22		

**Table 5:** Multiple linear regression results of screeners’ mastery of neonatal CHD screening knowledge (*n* = 2374).

Item	B Value	SE Value	β Value	*t*	*p*	95.0% Confidence Interval for B Value	
						Lower Limits	Upper Limits
constant	41.166	0.944	-	43.605	<0.001	39.315	43.017
occupation	−0.680	0.406	−0.035	−1.673	0.094	−1.477	0.117
department	−1.298	0.162	−0.171	−8.007	<0.001	−1.616	−0.980
seniority	0.525	0.185	0.088	2.839	0.005	0.162	0.888
professional title	−0.089	0.238	−0.012	−0.376	0.707	−0.555	−0.377

## Data Availability

The datasets used and analyzed in this study are available from the corresponding author on reasonable request.

## Abbreviations

CHD: congenital heart disease; CCHD: critical congenital heart disease; POX: pulse oximetry; MUR: murmur; PPH: Pan-pan Huang; QG: Qing Gu; XTZ: Xiao-ting Zhu; LLL: Li-ling Li; XJH: Xiao-jing Hu; GYH: Guo-ying Huang
